# Dynamic assessment of myocardial contractile dysfunction and its recovery after IVIG treatment in a murine model of Kawasaki disease using high-resolution speckle-tracking echocardiography

**DOI:** 10.3389/fcvm.2026.1695337

**Published:** 2026-03-04

**Authors:** Haiyong Wang, Zhiming Han, Yushan Zhou, Xin Dong, Nan Wang

**Affiliations:** The Department of Ultrasound Medicine, Gansu Provincial Hospital, Gansu, China

**Keywords:** Kawasaki disease, mouse model, myocardial function, speckle tracking echocardiography, strain analysis

## Abstract

**Background:**

Myocarditis is a common feature of acute Kawasaki disease (KD) and a major contributor to myocardial contractile dysfunction, which can be alleviated by timely intravenous immunoglobulin (IVIG) treatment. However, the effects of KD on myocardial systolic function and the impact of IVIG on myocardial recovery are not well understood in animal models. This study aims to explore whether left ventricular systolic dysfunction occurs in a KD mouse model and to evaluate the potential benefits of IVIG in mitigating myocardial contractile impairment using high-resolution speckle-tracking imaging (STI).

**Methods:**

We utilized a *Lactobacillus* casei cell-wall extract (LCWE)-induced murine model of KD vasculitis to assess the effects of IVIG treatment on myocardial dysfunction. Histological analyses and speckle-tracking strain imaging were performed to evaluate myocardial function during the progression of KD-induced vasculitis and myocarditis.

**Results:**

IVIG treatment significantly prevented both myocarditis and vasculitis. Conventional echocardiographic analyses showed differences in ejection fraction between the KD and control groups 14 days after LCWE injection, regardless of IVIG treatment. Notably, both the KD and KD + IVIG groups exhibited reduced longitudinal strain (LS) as early as 3 days post-injection compared to the control group. While LS remained decreased in the KD group throughout the disease progression, the KD + IVIG group showed a recovery to normal LS levels by day 56. At 14 and 28 days post-LCWE injection, LS in the KD group was significantly lower than in the KD + IVIG group. LS was negatively related to myocarditis scores (r = −0.94, *P* < 0.001).

**Conclusions:**

Myocardial contractile dysfunction resulting from myocarditis occurs in the KD mouse model and can be improved with IVIG treatment. High-resolution STI offers a more sensitive and accurate method for assessing myocardial dysfunction and the effects of cardioprotective treatments compared to conventional echocardiography.

## Introduction

Kawasaki disease (KD) is the leading reason of children acquired coronary artery disease in developed countries, and the etiology is still unclear until now (1, 2). Although characterized by acute systemic arteritis, the formation of coronary artery aneurysm (CAA) is the most severe pathological damage (1). KD patients with CAA are at risk of ischemic heart disease, myocardial infarction and even death. The occurrence of CAA is up to 20%–25% in untreated patients, however, this rate is reduced to 5% after timely high-dose intravenous immunoglobulin (IVIG) treatment (3).

Although most of the attention from KD clinical practice has been focused on the CAA and subsequent complications, KD-induced myocarditis is a far more universal feature than CAA (4–6). Histologic studies have shown that myocardial inflammatory injury is prevalent in patients with KD regardless of the presence of CAA (7, 8). Indeed, myocardial inflammatory cell infiltration and myocardial edema in KD patients have been already present prior to CAA formation (8). Myocardial contractile dysfunction existed in KD patients during the acute phase which responds readily to IVIG treatment (9).

*Lactobacillus* casei cell-wall extract (LCWE) induced vasculitis and coronary arteritis in KD mouse is the most mature and widely used animal model, which can histopathologically mimics pathological features of the cardiovascular lesions including coronary arteritis, coronary artery stenosis, aortitis, myocarditis and aneurysms seen in human KD (10–13). However, researches on this murine model of KD were mainly limited to pathology, immunology and molecular biology. Whether myocardial contractile dysfunction existed in this experimental model is not known. In addition, whether the probable myocardial dysfunction can be improved by IVIG treatment is also not fully explored.

Echocardiography has proven to be a useful tool for evaluating cardiac function in experimental animals (14–17). However, the sensitivity of conventional echocardiography in detecting subtle variations of LV performance is low due to its dependence on LV geometry assumption, and changes of conventional echocardiographic parameters often reveals late manifestations of the disease. High-resolution speckle tracking imaging (STI) is a novel imaging technique that enables accurately to evaluate global and regional myocardial function in laboratory animals with high reproducibility and feasibility (18–21). In addition, this valuable technique is angle independent and can detect subtle changes in myocardial performance (19, 22, 23).

In this study, we aimed to explore the characteristics of LV myocardial systolic function change in this mouse model of KD during different disease phases using high-resolution STI. Additionally, we also intended to explore the value of IVIG for improving probable myocardial contractile function abnormality caused by myocardial inflammation.

## Materials and methods

### Animals

Four-week-old male C57BL/6 mice were obtained from Lanzhou Veterinary Research Institute, Chinese Academy of Agricultural Sciences and housed under specific pathogen-free (SPF) conditions at the Department of Laboratory Animals, Gansu Provincial Hospital. All animal procedures were approved by the Institutional Animal Care and Use Committee of Gansu Provincial Hospital.

### Preparation of LCWE

LCWE was prepared following the method described by Noval Rivas et al. (11). Briefly, *Lactobacillus* casei, grown in MRS broth (Oxoid, UK) for 48 h, was harvested, washed with phosphate-buffered saline (PBS), and disrupted using 4% SDS. To remove any adherent material, sequential incubations with 250 µg/mL RNase, DNase I, and trypsin were performed. The cell wall fragments were washed with PBS, sonicated in a dry ice-ethanol bath for 2 h, and then centrifuged (20,000 g for 20 min). The supernatant was further centrifuged (95,000 g for 1 h at 4 °C), and the pellet was discarded. The total rhamnose content of the extract was measured using the phenol-sulfuric acid assay.

### KD mouse model and treatment protocols

Mice were randomly assigned to three groups: KD, KD + IVIG, and normal control, with 40 mice per group. In the KD and KD + IVIG groups, a single intraperitoneal injection of 0.5 mL LCWE (1 mg/mL) was administered to induce KD vasculitis. The control group received PBS. On day 5, the KD + IVIG group received intraperitoneal IVIG (2 g/kg; Lanzhou Bio, China).

### Histological evaluation

After echocardiographic examinations on days 3, 14, 28, and 56, five mice from each group were sacrificed by cervical dislocation. Cardiac tissues were harvested, embedded in optimal cutting temperature compound, and analyzed histologically by hematoxylin and eosin (HE) staining. Vessel inflammation in the aortic root and coronary artery was scored as follows: 0 = no inflammation, 1 = rare inflammatory cells, 2 = scattered inflammatory cells, 3 = diffuse inflammatory cell infiltrate, and 4 = dense inflammatory cell clusters. Myocarditis reflected by severity extent of myocardial involvemen was graded: 0 = no inflammation, 1 = >25% involvement, 2 = 25%–50% involvement, 3 = 50%–75% involvement, and 4 = >75% involvement. All histological evaluations and scoring were performed by an experienced investigator blinded to the experimental groups.

### Conventional echocardiography

Cardiac function was assessed using a high-resolution small animal ultrasound system (Vevo 3100, FUJIFILM VisualSonics, Toronto, ON, Canada) with a 22–55 MHz transducer. Mice were anesthetized with 2.5% isoflurane mixed with 0.5 L/min 100% O2. Hair removal cream was applied to the chest to expose the area from the neckline to mid-chest. Mice were positioned supine on a temperature-controlled operating table (37 °C) and maintained under steady-state anesthesia (1.0% isoflurane with 0.5 L/min 100% O2). Electrocardiogram and respiratory rate were monitored during imaging, and body temperature was maintained via a rectal probe. Dynamic images of three consecutive cardiac cycles in both parasternal long-axis and short-axis views were captured for strain analysis. To optimize image quality, depth, width, gain, and frame rate (233 Hz) were adjusted. LV end-diastolic and end-systolic diameters, inter-ventricular septum thickness, LV posterior wall thickness, left ventricular ejection fraction (LVEF), and left ventricular shortening fraction (LVSF) were obtained from grayscale M-mode images acquired in the parasternal short-axis view at the level of the papillary muscles, in accordance with established murine echocardiographic protocols (16). Measurements were averaged over three consecutive cardiac cycles. All conventional echocardiographic parameter analyses were conducted by a trained investigator blinded to the experimental groups.

### Speckle tracking-based strain analysis

Myocardial strain is defined as the change in myocardial fiber length relative to its original length, while strain rate (SR) refers to the rate of change in strain, expressed in 1/s (24). Strain and SR were measured in the radial, longitudinal, and circumferential directions based on myocardial fiber orientation and cardiac movement, using LV parasternal short- and long-axis views.

**Figure 1:**
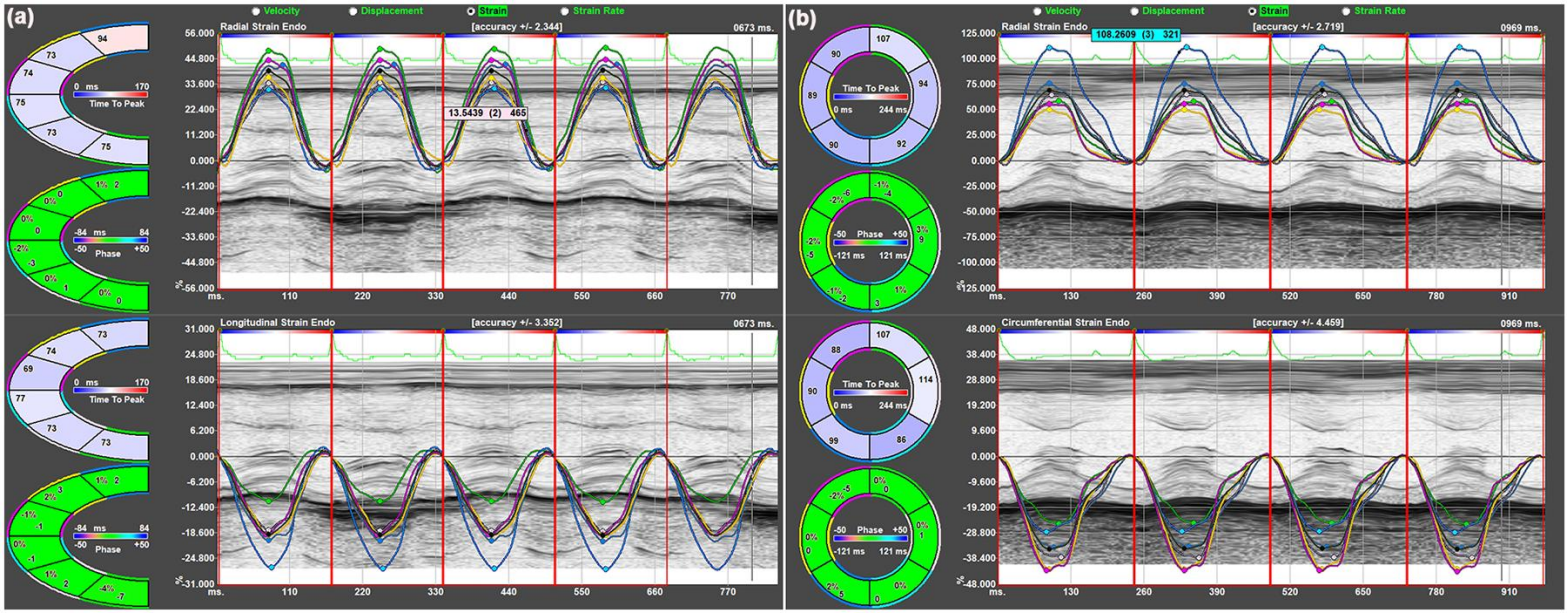
Speckle-tracking-based strain analysis in the left ventricle. **(a)** Segmental and global longitudinal strain, as well as radial strain, were derived from the long-axis view of the left ventricle. **(b)** Circumferential and radial strain were obtained from the short-axis view of the left ventricle.

In murine models, parasternal long-axis imaging provides reliable alignment with the left ventricular long axis while minimizing foreshortening and maintaining optimal spatial and temporal resolution. Therefore, longitudinal strain (LS) representing the percentage change in the length of the ventricle, was typically measured from the endocardial wall in the long-axis view (Figure 1a), which represents the most feasible and reproducible approach for evaluating longitudinal myocardial function in mice. Radial strain (RS) represents the percentage change in myocardial wall thickness and can be measured in both the short- and long-axis views, indicating myocardial deformation toward the center of the LV cavity (Figures 1a,b). Circumferential strain (CS), measured from the short-axis view of the LV, reflects the percentage change in myocardial fiber shortening along the circular perimeter of the heart (Figure 1b).

Digital B-mode images from three consecutive cardiac cycles were imported into the VevoStrain analysis workstation (VisualSonics, Toronto, ON, Canada). The endocardium and epicardium were manually traced to encompass the entire myocardial wall. If the initial trace was unsatisfactory, manual adjustments were made to obtain an accurate speckle tracking image. Strain and SR were assessed for six myocardial segments per LV view, with eight points measured in each segment, totaling 48 data points. The strain and SR values of each segment were averaged to provide the global systolic function of the myocardium. All strain analyses were conducted by the same trained investigator conducting conventional echocardiographic analysis who blinded to the experimental groups.

### Statistical analysis

Data are presented as mea*n* ± SD or mean ± SEM as appropriate. Strain and SR parameters are shown as absolute values to facilitate statistical comparison between groups. Differences between groups were assessed using one-way analysis of variance (ANOVA) or two-way ANOVA with post-test analysis for multiple group comparisons involving one or two independent variables, respectively. Spearman's coefficient was used for the correlation of myocardial strain and myocarditis scores. A *P*-value of <0.05 was considered statistically significant. All statistical analyses were performed using GraphPad Prism software version 5.00 (San Diego, CA, USA).

## Results

### Vasculitis and myocardial inflammation in the KD mouse model and response to IVIG

**Figure 2:**
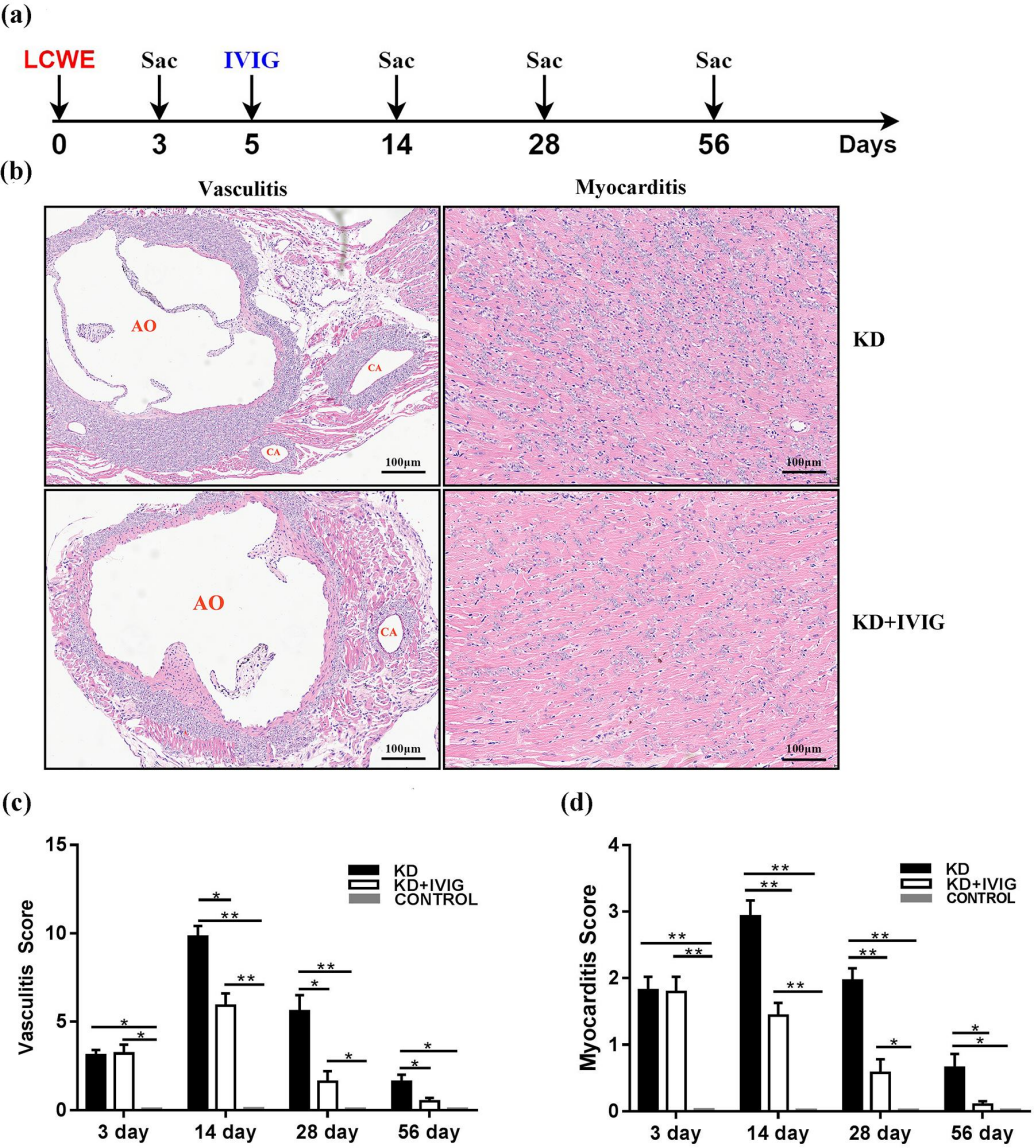
IVIG suppresses vasculitis and myocarditis in a murine model of KD induced by LCWE. **(a)** Schematic representation of the experimental design. Mice were injected with either LCWE alone or LCWE combined with IVIG administered on day 5 post-LCWE injection. Heart tissues were harvested at specified time points, and vascular and myocardial inflammation were assessed using hematoxylin and eosin (H&E) staining. **(b)** Representative H&E-stained sections of vascular and myocardial tissues from LCWE-injected mice with and without IVIG treatment at 14 days post-LCWE injection. Scale bars = 100 µm. **(c)** Quantitative vascular inflammation scores for LCWE-injected mice with or without IVIG treatment, as well as normal control mice. **(d)** Quantitative myocardial inflammation scores for LCWE-injected mice with or without IVIG treatment, as well as normal control mice. Data are presented as mean ± SEM. *P < 0.05, **P < 0.001*. LCWE: *Lactobacillus* casei cell-wall extract; IVIG: intravenous immunoglobulin; KD: Kawasaki disease.

The progression of LCWE-induced myocarditis and vasculitis in mice was evaluated at various time points: day 0 (prior to LCWE injection), and days 3, 14, 28, and 56 post-injection (Figure 2a). In the LCWE group, pronounced panvasculitis and myocardial inflammation were observed at all time points, with inflammation decreasing over time (Figures 2b–d).

In the LCWE + IVIG group, mice displayed similar pathological changes in the vasculature and myocardium prior to IVIG treatment, compared to the LCWE group (Figure 2b). However, after IVIG administration, inflammatory cell infiltration in the aortic root, coronary artery, and myocardium was reduced, with lower scores for both vasculitis (Figure 2c) and myocarditis (Figure 2d) in the LCWE + IVIG group compared to the LCWE group. These results confirm that the KD mouse model, consistent with clinical practice, was successfully established, and IVIG treatment effectively reduced inflammation. No inflammation was observed in the normal control group at any time point.

### Conventional echocardiographic findings

**Figure 3:**
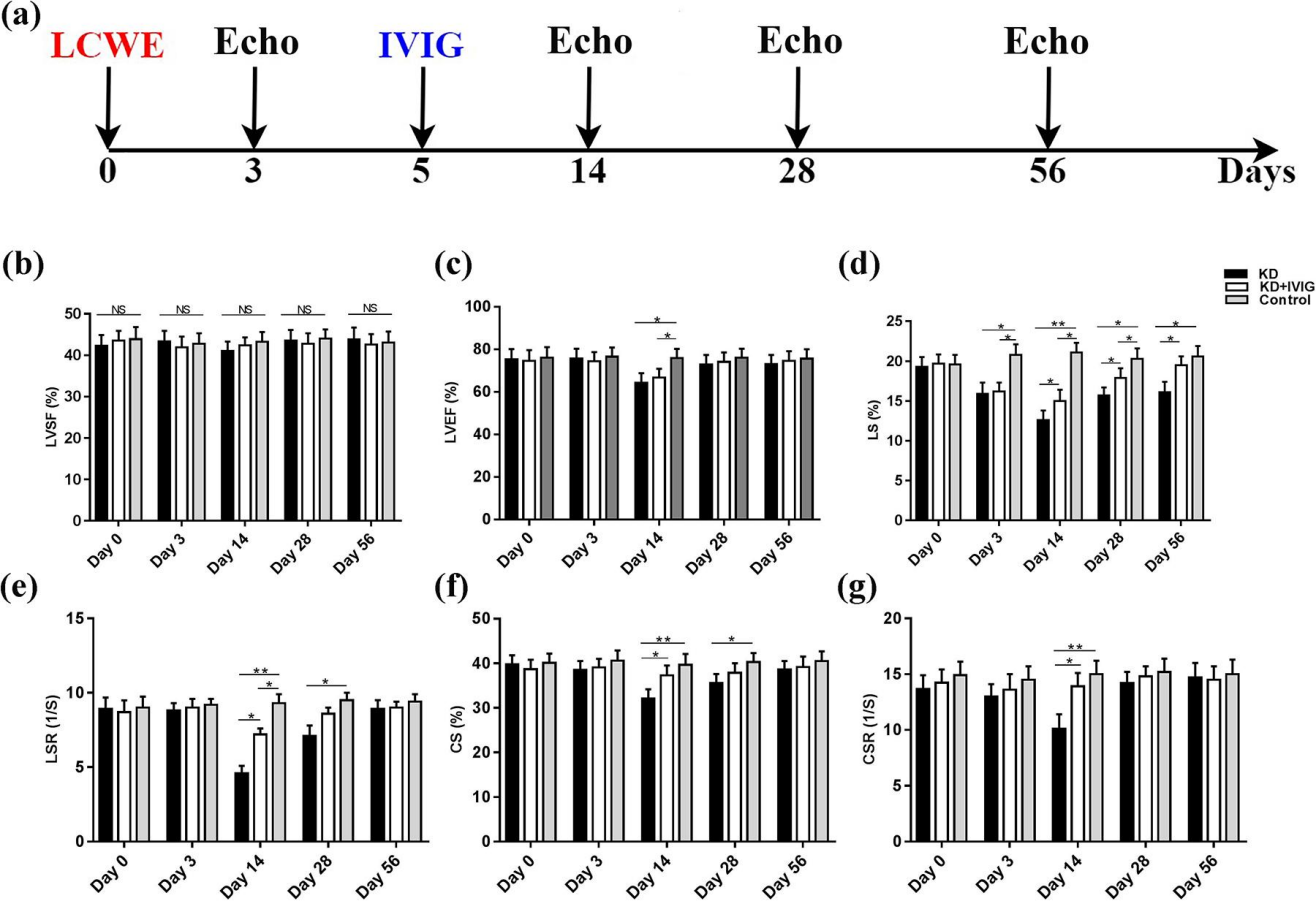
Assessment of left ventricular systolic function using M-mode echocardiography and high-resolution speckle-tracking imaging. **(a)** Schematic representation of the experimental design. **(b–g)** Comparison of systolic parameters among untreated KD mice, IVIG-treated KD mice, and normal controls, including LVEF, LVSF, LS, LSR, CS, and CSR at specified time points. Values are expressed as means ± SD, and myocardial strain and strain rate are presented as absolute values to facilitate statistical analysis. Data are presented as mean ± SD. *P < 0.05, **P < 0.001*. Echo: echocardiography; LVEF: left ventricular ejection fraction; LVSF: left ventricular shortening fraction; LS: longitudinal strain; LSR: longitudinal strain rate; CS: circumferential strain; CSR: circumferential strain rate.

To assess changes in LV systolic function, echocardiograms were performed before LCWE injection (day 0) and at days 3, 14, 28, and 56 post-injection (Figure 3a). Data collected from M-mode images are presented in Tables 1, 2. On day 0, no significant differences in conventional parameters were observed among the KD, KD + IVIG, and normal control groups (all *P* > 0.05) (Table 1). At day 14, both the KD and KD + IVIG groups showed decreased LVEF compared to the normal controls (all *P* < 0.05), but LVEF returned to normal by day 28 (Figure 3c).

**Table 1:** Characteristics of conventional echocardiography and high-resolution speckle-tracking echocardiography at baseline.

Variable	KD	KD + IVIG	Control	P
HR (bpm)	426 ± 18	431 ± 16	420 ± 14	0.67
BW(g)	18.12 ± 0.42	17.96 ± 0.38	18.26 ± 0.36	0.89
M-mode				
IVSd (mm)	0.69 ± 0.05	0.67 ± 0.06	0.66 ± 0.06	0.75
LVPWd (mm)	0.71 ± 0.04	0.69 ± 0.06	0.72 ± 0.04	0.77
LVIDd (mm)	3.2 ± 0.1	3.1 ± 0.1	3.1 ± 0.09	0.79
LVIDs (mm)	1.1 ± 0.03	1.2 ± 0.02	1.2 ± 0.03	0.65
LVEDV (µL)	33.5 ± 3.1	34.1 ± 3.0	33.5 ± 3.1	0.73
LVESV (µL)	6.3 ± 1.0	6.2 ± 0.08	6.1 ± 1.0	0.62
LVEF (%)	75.3 ± 4.8	74.6 ± 5.0	76.1 ± 4.9	0.59
LVSF (%)	42.3 ± 2.6	43.5 ± 2.4	43.9 ± 2.9	0.63
STE				
LAX-view				
LS (%)	19.3 ± 1.2	19.7 ± 1.1	19.6 ± 1.2	0.66
LSR (1/s)	8.9 ± 0.8	8.7 ± 0.7	9.0 ± 0.7	0.83
RS (%)	21.7 ± 1.3	20.8 ± 1.2	21.0 ± 1.2	0.29
RSR (1/s)	10.5 ± 0.4	10.8 ± 0.5	10.7 ± 0.4	0.59
SAX-view				
CS (%)	39.8 ± 2.0	38.7 ± 2.1	40.9 ± 2.1	0.77
CSR (1/s)	13.6 ± 1.2	14.2 ± 1.1	14.9 ± 1.2	0.65
RS (%)	34.7 ± 2.6	35.6 ± 2.2	35.1 ± 2.5	0.52
RSR (1/s)	11.6 ± 0.3	11.4 ± 0.3	12.0 ± 0.4	0.81

Values are presented as means ± SD, myocardial strain and strain rate are expressed as absolute values. HR: heart rate; BW: body weight; IVSd: diastolic interventricular septum; diastolic left ventricular posterior wall; LVIDd: diastolic left ventricular internal dimensions; LVIDs: systolic left ventricular internal dimensions; LVEDV: left ventricular end diastolic volume; LVESV: left ventricular end systolic volume; LVEF: left ventricular ejection fraction; LVSF: left ventricular shortening fraction; LVM: left ventricular mass; STE: speckle-tracking echocardiography; LAX: long axial; SAX: short axial; LS: longitudinal strain; LSR: longitudinal strain rate; RS: radial strain; RSR: radial strain rate; CS: circumferential strain; CSR: circumferential strain rate.

**Table 2:** Conventional echocardiographic data in the studied mice at 3, 14, 28 and 56 days after treatment of LCWE.

Variable	3 day			14 day			28 day			56 day		
	KD	KD + IVIG	Control	KD	KD + IVIG	Control	KD	KD + IVIG	Control	KD	KD + IVIG	Control
HR (bpm)	431 ± 21	434 ± 17	426 ± 19	430 ± 18	427 ± 17	424 ± 16	428 ± 18	429 ± 15	425 ± 17	429 ± 16	428 ± 17	431 ± 17
IVSd (mm)	0.65 ± 0.04	0.63 ± 0.03	0.64 ± 0.04	0.66 ± 0.05	0.68 ± 0.03	0.65 ± 0.04	0.71 ± 0.05	0.68 ± 0.03	0.67 ± 0.04	0.71 ± 0.04	0.69 ± 0.02	0.68 ± 0.03
LVPWd (mm)	0.67 ± 0.02	0.64 ± 0.05	0.65 ± 0.03	0.69 ± 0.04	0.70 ± 0.05	0.63 ± 0.03	0.70 ± 0.03	0.68 ± 0.04	0.65 ± 0.05	0.72 ± 0.02	0.70 ± 0.03	0.68 ± 0.03
IVSd/LVPWd	1.0 ± 0.02	0.9 ± 0.01	0.9 ± 0.02	1.0 ± 0.02	0.9 ± 0.03	1.0 ± 0.02	0.9 ± 0.03	0.9 ± 0.01	1.0 ± 0.03	0.8 ± 0.02	0.9 ± 0.02	0.9 ± 0.03
LVIDd (mm)	3.2 ± 0.1	3.1 ± 0.1	3.2 ± 0.1	3.2 ± 0.09	3.1 ± 0.1	3.1 ± 0.1	3.2 ± 0.1	3.1 ± 0.1	3.2 ± 0.09	3.2 ± 0.1	3.2 ± 0.09	3.1 ± 0.1
LVIDs (mm)	1.1 ± 0.04	1.2 ± 0.03	1.2 ± 0.04	1.1 ± 0.02	1.2 ± 0.03	1.1 ± 0.02	1.1 ± 0.04	1.2 ± 0.03	1.1 ± 0.03	1.1 ± 0.03	1.2 ± 0.04	1.2 ± 0.03
LVEDV (µL)	35.0 ± 2.9	34.8 ± 3.1	34.6 ± 3.1	35.6 ± 3.0	35.0 ± 3.2	34.9 ± 2.9	34.2 ± 3.3	35.4 ± 3.1	35.0 ± 3.1	35.4 ± 3.1	35.0 ± 3.1	35.2 ± 3.2
LVESV (µL)	6.5 ± 0.98	6.3 ± 1.0	6.4 ± 1.2	6.5 ± 1.1	6.4 ± 1.1	6.4 ± 1.2	6.5 ± 1.2	6.4 ± 1.0	6.3 ± 1.0	6.4 ± 1.1	6.5 ± 1.2	6.4 ± 1.0
LVEF (%)	75.7 ± 4.6	74.4 ± 4.3	76.5 ± 4.4	64.3 ± 4.5*	66.8 ± 4.1*	75.9 ± 4.3	72.9 ± 4.5	74.1 ± 4.4	76.0 ± 4.3	73.1 ± 4.3	74.6 ± 4.5	75.6 ± 4.4
LVSF (%)	43.4 ± 2.5	41.9 ± 2.6	42.8 ± 2.5	41.0 ± 2.3	42.4 ± 1.9	43.2 ± 2.4	43.5 ± 2.9	42.8 ± 2.5	44.0 ± 2.2	43.8 ± 2.9	42.6 ± 2.5	43.1 ± 2.6

Values are presented as means ± SD, *P < 0.05 vs. Control. HR: heart rate; IVSd: diastolic interventricular septum; diastolic left ventricular posterior wall; LVIDd: diastolic left ventricular internal dimensions; LVIDs: systolic left ventricular internal dimensions; LVEDV: left ventricular end diastolic volume; LVESV: left ventricular end systolic volume; LVEF: left ventricular ejection fraction; LVSF: left ventricular shortening fraction; LCWE: lactobacillus casei cell-wall extract, KD: Kawasaki disease; IVIG: intravenous immunoglobin.

### Myocardial contractile performance assessed by STI

Table 3 presents the strain analysis data comparing the three groups throughout the study. As early as day 3 post-LCWE injection, both the KD and KD + IVIG groups showed significantly decreased LS compared to the normal control group, and the reduced LS in the KD group persisted throughout the study (Figure 3d). At day 14, LS, longitudinal strain rate (LSR), CS, and circumferential strain rate (CSR) were significantly decreased in the KD group compared to normal controls (all *P* < 0.001). In contrast, the KD + IVIG group only showed lower LS and LSR compared to controls (all *P* < 0.05) (Figures 3d–g). Additionally, the KD group exhibited more pronounced reductions in LS, LSR, CS, and CSR compared to the KD + IVIG group (Figures 3d–g). By day 28, LS, LSR, and CS in the KD group remained decreased compared to the control group, while the KD + IVIG group showed improved LS compared to the KD group (Figures 3d–f). At day 56, LS remained decreased in the KD group compared to both the KD + IVIG and normal control groups (all *P* < 0.05) (Figure 3d).

**Table 3:** Speckle-tracking based echocardiographic data in the studied mice at 3, 14, 28 and 56 days after treatment of LCWE.

Variable	3 day			14 day			28 day			56 day		
	KD	KD + IVIG	Control	KD	KD + IVIG	Control	KD	KD + IVIG	Control	KD	KD + IVIG	Control
LAX-view												
LS (%)	15.9 ± 1.4*	16.2 ± 1.1*	20.8 ± 1.3	12.6 ± 1.2**,^†^	15.0 ± 1.4*	21.1 ± 1.2	15.7 ± 1.0*^,^^†^	17.9 ± 1.2*	20.3 ± 1.3	17.4 ± 1.3*^,^^†^	19.8 ± 1.1	20.6 ± 1.3
LSR (1/s)	8.8 ± 0.5	9.0 ± 0.6	9.2 ± 0.4	4.6 ± 0.5**^,^^†^	7.2 ± 0.4*	9.3 ± 0.6	7.1 ± 0.7*	8.6 ± 0.4	9.5 ± 0.5	8.9 ± 0.6	9.0 ± 0.4	9.4 ± 0.5
RS (%)	19.8 ± 1.1	20.2 ± 1.0	21.3 ± 1.2	20.0 ± 1.2	20.9 ± 1.1	22.4 ± 1.2	20.6 ± 1.1	21.2 ± 1.2	21.0 ± 1.1	21.5 ± 1.0	21.2 ± 1.1	22.1 ± 1.0
RSR (1/s)	8.9 ± 0.37	9.5 ± 0.39	10.6 ± 0.42	9.3 ± 0.3	9.7 ± 0.4	10.4 ± 0.4	10.0 ± 0.3	9.8 ± 0.3	10.3 ± 0.4	10.0 ± 0.3	9.8 ± 0.3	10.3 ± 0.4
SAX-view												
CS (%)	38.5 ± 2.0	39.1 ± 1.9	40.6 ± 2.3	32.1 ± 2.1**^,^^†^	37.3 ± 2.2	39.7 ± 2.4	35.6 ± 2.0*	37.9 ± 2.1	40.3 ± 2.0	38.6 ± 1.9	39.2 ± 2.3	40.5 ± 2.2
CSR (1/s)	13.0 ± 1.1	13.6 ± 1.4	14.5 ± 1.2	10.1 ± 1.3**^,^^†^	13.9 ± 1.2	15.0 ± 1.2	14.2 ± 1.0	14.8 ± 0.9	15.2 ± 1.2	14.7 ± 1.3	14.5 ± 1.2	15.0 ± 1.3
RS (%)	32.6 ± 2.3	33.7 ± 2.4	35.9 ± 2.4	32.9 ± 2.3	34.0 ± 2.2	34.8 ± 2.2	34.7 ± 2.2	35.5 ± 2.3	35.2 ± 2.3	34.3 ± 2.1	35.6 ± 2.3	35.9 ± 2.1
RSR (1/s)	9.4 ± 0.2	11.1 ± 0.3	12.2 ± 0.3	10.9 ± 0.2	12.1 ± 0.3	12.7 ± 0.2	11.8 ± 0.2	11.6 ± 0.3	12.3 ± 0.3	12.0 ± 0.3	11.9 ± 0.3	12.5 ± 0.2

Values are presented as means ± SD, myocardial strain and strain rate are expressed as absolute values. *P < 0.05 vs. Control, **P < 0.001 vs. Control, ^†^*P* < 0.05 vs. KD + IVIG. STE: speckle-tracking echocardiography; LAX: long axial; SAX: short axial; LS: longitudinal strain; LSR: longitudinal strain rate; RS: radial strain; RSR: radial strain rate; CS: circumferential strain; CSR: circumferential strain rate, LCWE: lactobacillus casei cell-wall extract; KD: Kawasaki disease; IVIG: intravenous immunoglobin.

**Figure 4:**
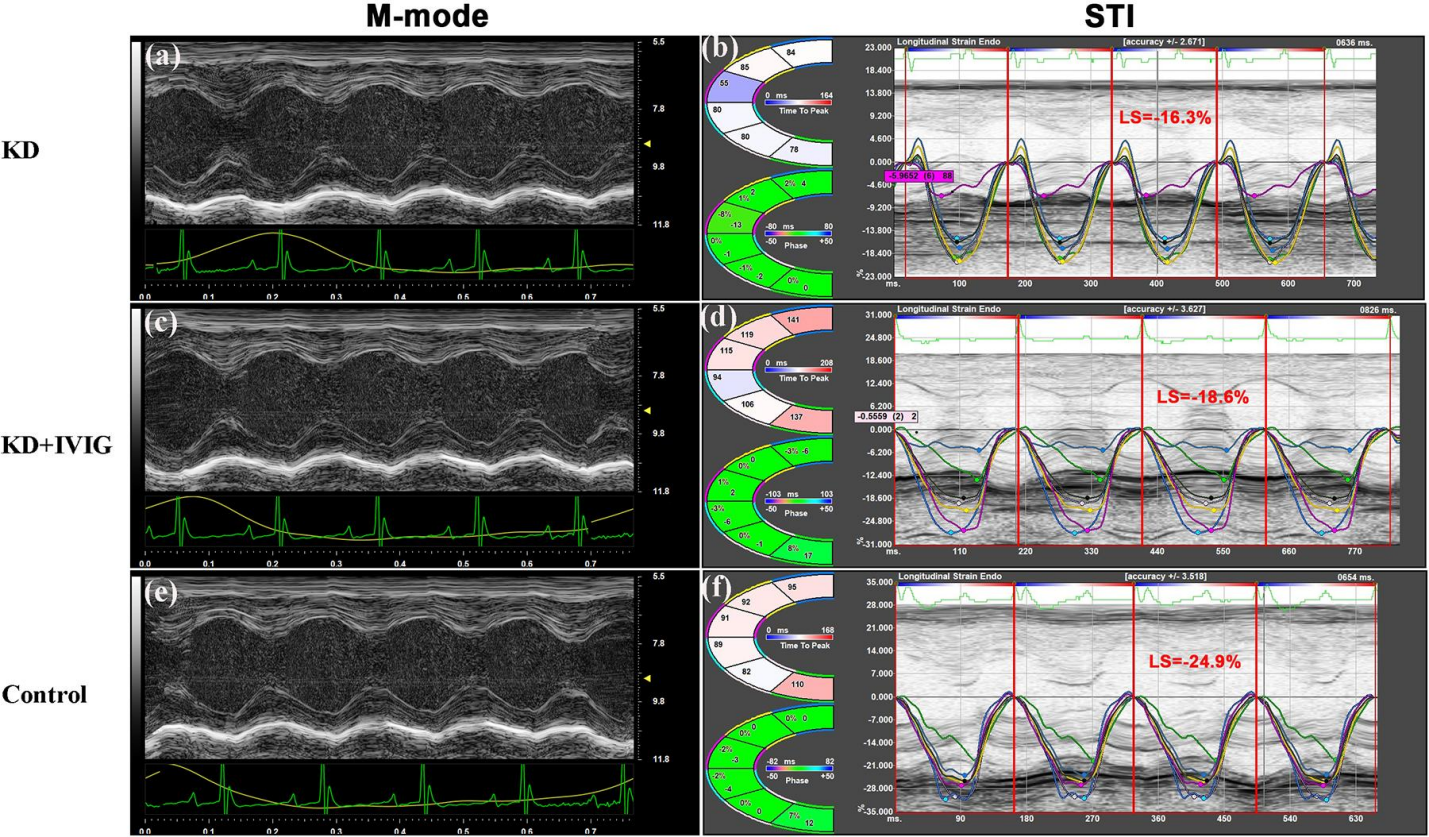
Representative images of M-mode echocardiography **(a,c,e)** and speckle-tracking-derived LS **(b,d,f)** in LCWE-treated, LCWE + IVIG-treated, and PBS-control groups at day 28 post-LCWE injection. Despite normal left ventricular systolic function as shown by M-mode echocardiography in all three groups, the LS in LCWE-treated mice (−14.5%) **(b)** was significantly reduced compared to LCWE + IVIG-treated mice (−18.7%) **(d)** and PBS controls (−19.3%) **(f)** Furthermore, LS values were comparable between LCWE + IVIG-treated mice and PBS controls. LS = longitudinal strain; KD = Kawasaki disease; IVIG=intravenous immunoglobulin; STI=speckle-tracking imaging.

Representative M-mode echocardiographic images and LS measurements from STI at day 28 post-LCWE injection are shown in Figure 4 for the KD, KD + IVIG, and PBS control groups.

### Correlation with myocarditis scores and myocardial strain

**Figure 5:**
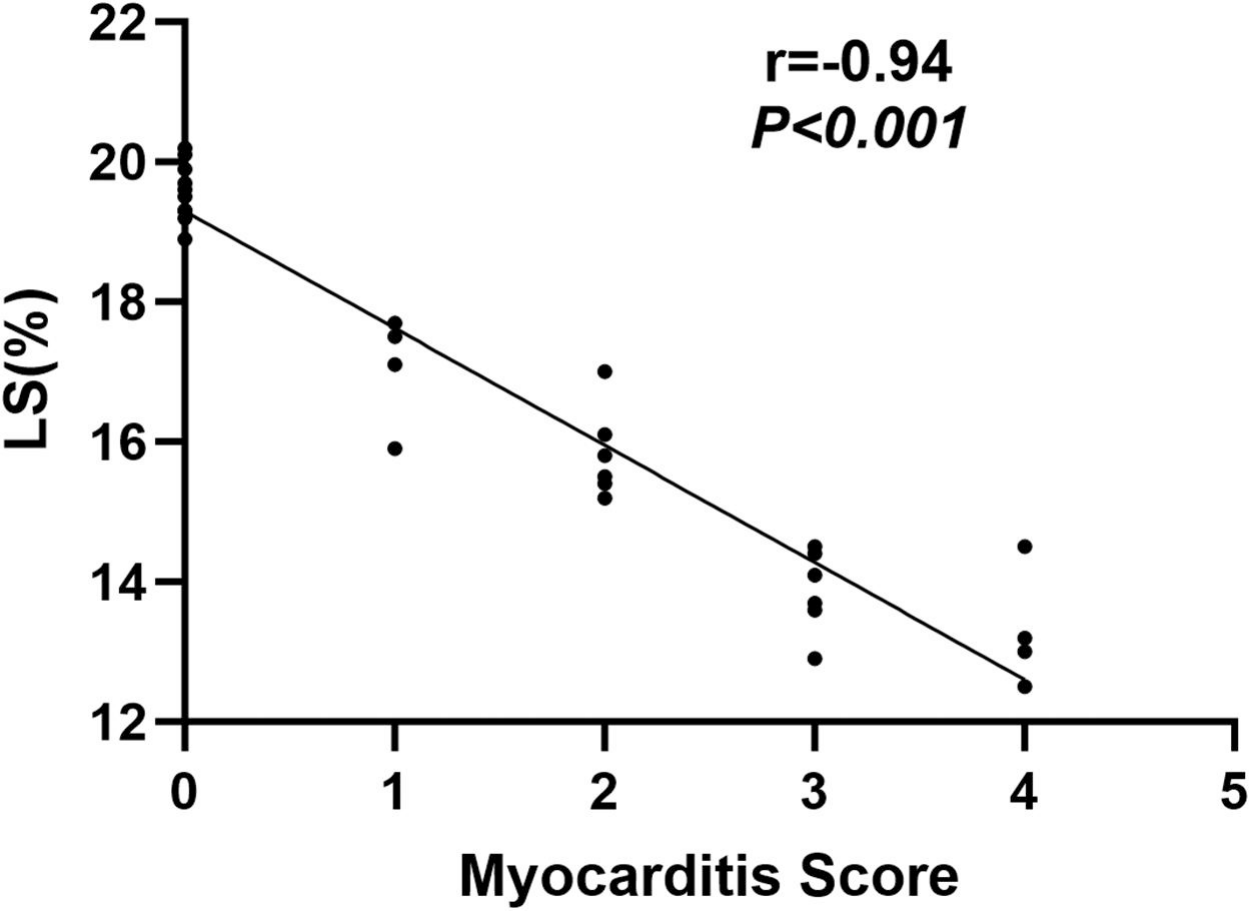
Correlations between LS and myocarditis score. LS is presented as absolute values to facilitate statistical analysis. LS = longitudinal strain.

LS was negatively related to myocarditis scores (r = −0.94, *P* < 0.001) (Figure 5), and no significant relationship between other deformation parameters derived from STI and myocarditis scores was found.

## Discussion

To the best of our knowledge, this study is the first to apply high-resolution STI for non-invasive early detection of LV myocardial contractile dysfunction in a KD mouse model. Additionally, we demonstrated the effectiveness of IVIG treatment in improving myocardial contractile function in this experimental model through strain-based analyses.

This study provides new insights into the evolution of myocardial contractile dysfunction in KD mice by combining cardiac histology with myocardial function assessment using high-resolution STI. The etiology and pathogenesis of KD remain unclear. Although much attention has been given to coronary artery abnormalities and their long-term complications, KD-induced myocarditis is a more ubiquitous histological finding in all patients, regardless of coronary involvement (4, 6, 7, 25). A study by Yutani et al. (26) found that myocardial abnormalities, including lymphocytic and plasma cell infiltration, myocardial fibrosis, and disarray of myocardial fibers, were present in all KD patients. Similarly, Harada et al. (8) noted that inflammatory cell infiltration was seen in the myocardial interstitium of all KD patients during the acute phase, even before the development of epicardial coronary arteritis. However, these studies relied on histopathological analysis, which is invasive and carries serious complications such as bleeding, arrhythmias, and sampling errors, limiting its clinical applicability (27, 28). Moreover, myocardial contractile function was not assessed using imaging modalities in these studies. In contrast, our study successfully established a KD mouse model characterized by vasculitis and myocardial inflammation, consistent with the pathological changes seen in KD patients, and demonstrated the efficacy of IVIG treatment. Additionally, we evaluated changes in myocardial systolic function during different stages of the disease, providing a valuable platform for assessing therapeutic strategies for improving cardiac function in KD.

High-resolution STI proved to be a more sensitive method for detecting early impairment of LV myocardial contractile function in the KD mouse model, compared to conventional echocardiographic measurements. LV systolic function, a key indicator of cardiac performance, is essential for diagnosing various cardiovascular diseases (29–31). According to the American Heart Association's revised guidelines for diagnosing KD, LV systolic dysfunction during the acute phase is a crucial diagnostic marker for incomplete KD (1). While conventional echocardiography is commonly used for assessing cardiac structure and function, it is less sensitive to subtle abnormalities that occur early in the disease progression. Changes in conventional parameters, such as LVEF, typically appear later in the disease, indicating more advanced dysfunction. In our study, we observed a decrease in LS as early as 3 days post-LCWE injection, prior to detectable changes in LVEF which remained unchanged until 14 days post-injection. LS in the KD group persisted at reduced levels throughout the disease course, further confirming that speckle-tracking strain analysis is a reliable tool for detecting early changes in LV myocardial function in the KD mouse model. These findings align with those of Shepherd et al. (32), who observed early changes in RS, CS, and LS in a type 1 diabetes mouse model before conventional echocardiography detected a decline in LVEF. Zhang et al. (32) found that conventional echocardiography failed to detect subtle myocardial dysfunction in the KD mouse model, supporting our conclusion that standard echocardiographic techniques are insufficient for detecting early myocardial changes.

In line with clinical treatment, IVIG treatment effectively mitigated myocarditis and coronary arteritis in the KD mice, as well as improved LV myocardial contractile function, as monitored by high-resolution STI. High-dose IVIG is the most effective clinical treatment for KD patients during the acute phase, significantly reducing the occurrence of CAAs and rapidly improving clinical symptoms (1, 3). However, the mechanisms by which IVIG improves vasculitis and myocarditis in KD patients remain unclear, though they may involve modulation of inflammatory cytokines, inhibition of superantigens, and regulation of immune cell functions (33). Takahashi et al. (34) demonstrated that IVIG suppressed the progression of vasculitis in a murine KD model, which is consistent with our findings. However, their study did not assess the impact of IVIG on myocardial systolic function. In our study, IVIG-treated KD mice exhibited less severe myocarditis and coronary arteritis compared to untreated KD mice. Additionally, no significant differences in speckle-tracking strain parameters were observed between IVIG-treated mice and normal controls at day 56, while LS remained lower in untreated KD mice, suggesting that persistent myocarditis contributes to myocardial dysfunction. Moreover, negative correlation between LS and myocarditis score was found which indicated that myocarditis may be the primary reason leading to myocardial dysfunction in KD mice. Based on these findings, our study further elucidates the mechanism of action of IVIG, suggesting that it enhances myocardial contractile function in KD mice by attenuating cardiac inflammatory injury—an effect consistent with clinical outcomes observed in patients with KD. Additionally, this mouse model, combined with high-resolution speckle-tracking analysis, provides valuable information for evaluating new drug therapies aimed at suppressing myocarditis and improving myocardial function.

Among the strain parameters, global LS demonstrated greater sensitivity than CS in detecting LV myocardial systolic dysfunction in the KD mice. Myocardial fibers are arranged in a helical and perpendicular orientation, with a predominance of circumferential fibers and fewer longitudinal fibers, particularly in the subendocardium (35). Due to the high load on longitudinal fibers during contraction and their relative susceptibility to injury, LS tends to decline earlier than CS and RS (36, 37). In our study, we observed a decrease in LS as early as 3 days post-LCWE injection, which was prior to the decline in CS at day 14. These findings suggest that LS is a more sensitive and reliable marker for detecting early myocardial dysfunction in KD than other strain parameters, and it provides a useful tool for rapid cardiac function assessment in preclinical studies.

### Limitations

Our study has several limitations. First, the sample size was relatively small, though the statistical differences in strain parameters were significant enough to support our conclusions. Second, speckle-tracking strain analysis was performed under anesthesia with isoflurane, which may have influenced cardiac function due to the suppression of heart rate. However, multiple studies have demonstrated that echocardiographic measurements, including speckle-tracking strain analyses, remain reliable even under anesthesia (16, 38–40). In our study, heart rates were maintained near physiological levels by adjusting the isoflurane concentration dynamically. Third, speckle-tracking and echocardiographic measurements were conducted by a single experienced operator blinded to the experimental groups. While this ensured analytical consistency, inter-observer and intra-observer variability were not formally assessed, which may represent a minor limitation with respect to measurement reproducibility. Future studies incorporating reproducibility analyses would further strengthen these findings. Fourth, assessment of longitudinal myocardial function was performed using parasternal long-axis imaging rather than apical views. Although apical imaging is preferred in human echocardiography (41–43), acquisition of true apical four- or two-chamber views in mice is frequently limited by thoracic anatomy, cardiac orientation, and the risk of ventricular foreshortening, which may compromise measurement accuracy. Current murine echocardiography guidelines emphasize accurate alignment with the left ventricular long axis and image quality rather than strict adherence to apical views (16). Accordingly, parasternal long-axis imaging has been widely adopted and validated for longitudinal strain assessment in murine studies (16, 18, 19). Lastly, variations in software algorithms across different vendors for strain analysis create challenges in comparing strain parameters (44, 45), which poses another inherent limitation.

## Conclusions

In the KD mouse model, similar to human KD, myocardial contractile dysfunction occurs and can be attenuated by IVIG treatment. High-resolution STI proves to be a promising approach for assessing cardioprotective agents aimed at reducing cardiovascular injury and improving myocardial function. This technique has the potential to significantly enhance the evaluation of cardiac phenotypes in KD models, supporting the use of high-resolution STI as a valuable echocardiographic modality for investigating myocardial dysfunction in KD mouse models.

## Data Availability

The raw data supporting the conclusions of this article will be made available by the authors, without undue reservation.
